# Targeted brain delivery of RVG29‐modified rifampicin‐loaded nanoparticles for Alzheimer's disease treatment and diagnosis

**DOI:** 10.1002/btm2.10395

**Published:** 2022-08-26

**Authors:** Ruiyi Zhou, Lihong Zhu, Zhaohao Zeng, Rixin Luo, Jiawei Zhang, Rui Guo, Lei Zhang, Qunying Zhang, Wei Bi

**Affiliations:** ^1^ Department of Neurology The First Affiliated Hospital, Jinan University Guangzhou People's Republic of China; ^2^ Department of Pathophysiology Key Laboratory of State Administration of Traditional Chinese Medicine of the People's Republic of China, School of Medicine, Jinan University Guangzhou People's Republic of China; ^3^ Key Laboratory of Biomaterials of Guangdong Higher Education Institutes Guangdong Provincial Engineering and Technological Research Center for Drug Carrier Development, Department of Biomedical Engineering, Jinan University Guangzhou People's Republic of China; ^4^ Department of Cerebrovascular Disease The Fifth Affiliated Hospital, Sun Yat‐sen University Zhuhai People's Republic of China; ^5^ Department of Cardiology The Fifth Affiliated Hospital, Sun Yat‐sen University Zhuhai People's Republic of China

**Keywords:** Alzheimer's disease, blood–brain barrier, brain targeting, MRI, rifampicin, β‐amyloid plaques

## Abstract

Alzheimer's disease (AD) is an aging‐related neurodegenerative disease. The main pathological features of AD are β‐amyloid protein (Aβ) deposition and tau protein hyperphosphorylation. Currently, there are no effective drugs for the etiological treatment of AD. Rifampicin (RIF) is a semi‐synthetic broad‐spectrum antibiotic with anti‐β‐amyloid deposition, anti‐inflammatory, anti‐apoptosis, and neuroprotective effects, but its application in AD treatment has been limited for its strong hydrophobicity, high toxicity, short half‐life, low bioavailability, and blood–brain barrier hindrance. We designed a novel brain‐targeted and MRI‐characteristic nanomedicine via loading rabies virus protein 29 (RVG29), rifampicin, and Gd on poly (l‐lactide) nanoparticles (RIF@PLA‐PEG‐Gd/Mal‐RVG29). The cytotoxicity assay demonstrated that RIF@PLA‐PEG‐Gd/Mal‐RVG29 had favorable biocompatibility and security. Fluorescence imaging in vivo showed that PLA‐PEG‐Gd/Mal‐RVG29 could deliver rifampicin into the brain by enhancing cellular uptake and brain targeting performance, leading to improvement of the bioavailability of rifampicin. In in vivo study, RIF@PLA‐PEG‐Gd/Mal‐RVG29 improved the spatial learning and memory capability of APP/PS1 mice in the Morris water maze, as compared to rifampicin. Immunofluorescence, TEM, immunoblotting, and H&E staining revealed that RIF@PLA‐PEG‐Gd/Mal‐RVG29 reduced Aβ deposition in hippocampal and cortex of APP/PS1 mice, improved the damage of synaptic ultrastructure, increased the expression level of PSD95 and SYP, as well as reduced the necrosis of neurons. These findings suggest that RIF@PLA‐PEG‐Gd/Mal‐RVG29 may be an effective strategy for the treatment of AD.

## 1 INTRODUCTION

Most neurodegenerative diseases are associated with progressively pathological accumulation of one or more protein aggregates.^1^, ^2^ Alzheimer's disease (AD) is one of the progressive and onset‐concealed neurodegenerative diseases that affect approximately 50 million population worldwide and is the primary cause of dementia. Previous studies showed that the aggregation of Aβ peptides may trigger the deposition of amyloid fibers in neural tissue, leading to atrophy and nerve cell death, which are one of the dominating factors in the pathogenesis of AD.^3^, ^4^ Therefore, effectively inhibiting the deposition of abnormal proteins is essential for the treatment of AD.

Rifampicin (RIF) is a semi‐synthetic broad‐spectrum antibiotic extracted from rifampicin B and is mainly used in the clinical treatment of tuberculosis and leprosy. It has protective effects in a variety of central nervous system (CNS) diseases, such as ischemic stroke,^5^, ^6^ Parkinson's disease,^7^, ^8^ multisystem atrophy,^9^ multiple sclerosis,^10^ meningitis^11^ and AD.^12^ RIF can reduce the proinflammatory mediator release, neuronal apoptosis, Aβ oligomers accumulation, and Tau protein hyperphosphorylation in AD mice.^13^, ^14^, ^15^, ^16^ Although RIF has definite potential efficacy on anti‐dementia, anti‐inflammatory, anti‐apoptosis, and neuroprotection, it occasionally causes severe side effects (such as liver damage). In addition, oral RIF has a very low utilization efficiency after intestinal absorption and first‐pass effects on the liver. Therefore, it is necessary to find a novel targeting drug delivery approach to avoid the first‐pass effect on the liver, and the side effects, that will achieve better therapeutic effects.

Although drug therapy has become an effective strategy in the treatment of AD and related neurodegenerative diseases. However, only a few drugs are available for the clinical treatment of AD. These traditional drugs may cause higher side effects on normal cells, and their therapeutic effect is still limited. Molecular chaperones were found to be effective inhibitors of neurodegenerative diseases due to their ability to inhibit intermolecular interactions between abnormal proteins.^17^ Recently, Huang et al. prepared a novel mixed‐shell polymeric micelles (MSPMs) artificial chaperone to efficiently inhibit the accumulation of Aβ and reduce neuronal cell cytotoxicity.^18^ The presence of the blood–brain barrier (BBB) is a major obstacle to treating neurodegenerative diseases, which prevents the access of nano‐drugs to the brain from the blood. In recent years, functional nanomaterials have been used in the treatment of neurodegenerative diseases due to their excellent biocompatibility and drug delivery properties. Encapsulating small drug molecules in nanomaterials can improve their delivery efficiency in the central nervous system.^19^, ^20^ Some targeted ligands, such as insulin and lactoferrin have been employed to carry therapeutic drugs into the brain.^21^, ^22^ Brain‐targeted polymer nanoparticles that can deliver drugs through the BBB are considered a promising strategy for the treatment of neurological diseases.^23^ These polymer nanoparticle drug delivery systems can carriage therapeutic drug molecules that would otherwise be impermeable to the BBB. In recent years, brain‐targeted drug delivery systems have been considered as potential therapeutics for AD.^24^, ^25^ However, the brain‐targeting properties and drug delivery efficiency of existing polymer drug delivery systems are still limited.

Brain‐targeted RVG29, a 29 amino acid peptide originating from the rabies virus glycoprotein (RVG), has a specific binding effect on the nicotinic acetylcholine receptors (nAchR) on neuronal cells.^26^ Because nAchR is widely present on the surface of neurons and capillary endothelial cells in the brain, RVG29 can effectively cross the BBB into the brain via nAchR‐mediated endocytosis.^27^ It has been demonstrated that biodegradable polyethylenimine modified with RVG peptide as targeting ligands for neuronal cells can promote gene delivery to the brain.^28^ Kumar et al. found that a chimeric peptide combined with nona(d‐arginine) peptide and RVG could bind and deliver siRNAs to the central nervous system, leading to objective gene silencing in the brain.^29^ So far, RVG29‐modified nano‐drug delivery systems, including nucleic acids,^30^ biologically derived or synthetic nanoparticles,^31^, ^32^, ^33^ nano‐device^27^, ^34^ and liposomes,^35^ can transport a variety of volume‐large drugs across the BBB and promote drug accumulation in the brain when administered systemically in the treatment of neurodegenerative diseases. Targeted drug delivery systems can not only enhance the efficiency of drug treatment of lesions, but also help reduce the side effects of drugs on normal cells. Therefore, RVG‐modified drug carriers provide a safe and non‐invasive potential therapeutic strategy for drug delivery across the BBB to treat neurological diseases.

Herein, we designed and constructed nanoparticles by assembling and synthesizing biodegradable polylactic acid, contrast‐enhanced gadolinium (Gd), and inflammation‐eliminated rifampicin. Then its surface was modified by targeting protein RVG‐29 to obtain a novel brain‐targeted nanomedicine RIF@PLA‐PEG‐Gd/Mal‐RVG29. This nanomedicine can penetrate the BBB of mice with AD disease to achieve targeted therapy, which is beneficial to alleviate brain inflammation, delay the pathological process of AD and improve the cognitive function of mice. In addition, the magnetic resonance imaging characteristics of Gd have been used to diagnose AD in vivo and evaluate the efficacy of nanomedicine, providing new strategies and methods for accurate diagnosis and treatment of AD. Currently, the potential application of RVG29‐modified delivery system combined brain‐targeted therapy, anti‐inflammatory, and magnetic resonance imaging functions for treatment and diagnosis of AD remains unexplored.

## 2 MATERIALS AND METHODS

### 2.1 Materials

Poly(l‐lactide)‐poly(ethylene glycol)‐maleimide (PLA‐PEG‐Mal, MW of PLA is 10 k and MW of PEG is 5k, >90%) and poly(l‐lactide)‐poly(ethylene glycol)‐amine (PLA‐PEG‐NH_2_, MW of PLA is 10k and MW of PEG is 5k, >90%) were purchased from Xi'an Ruixi Biotechnology Co., Ltd. Rifampicin (98%), triethylamine, dimethyl sulfoxide, dioxane, and gadolinium chloride hexahydrate (98%) were purchased from Shanghai Macklin Biochemical Technology Co., Ltd. Diethylenetriaminepentaacetic acid (DTPA, 98%) was bought from Aladdin. RVG29 (YTIWMPENPRPGTPCDIFTNSRGKRASNGC) was purchased from Shanghai Apeptide Co., Ltd. Tetrahydrofurfuryl chloride was bought from Tianjin Damao Chemical Reagent Factory. Other chemicals were analytical‐grade level, and all the aqueous solutions were prepared by using DI water. The Aβ antibody (1–42 specific), PSD95 antibody, SYP antibody, and beta‐Tubulin antibody were purchased from Cell Signaling Technology. The horseradish peroxidase‐conjugated secondary antibody was purchased from Beijing Dinguo Changsheng Biotechnology Co., Ltd. The TRITC donkey anti‐rabbit secondary antibody was purchased from Life Technologies (Thermo Fisher Scientific).

### 2.2 Preparation of RIF@PLA‐PEG‐Gd/Mal and RIF@PLA‐PEG‐Gd/Mal‐RVG29

**SCHEME 1:**
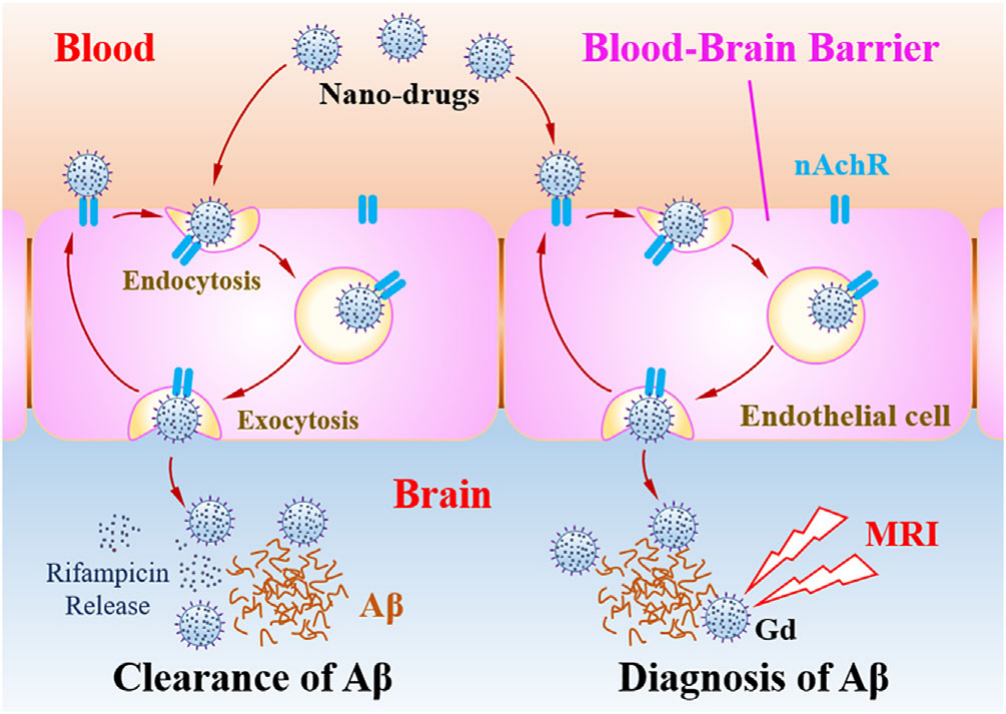
Brain‐targeted nanomedicine to improve the clearance of β‐amyloid deposition a of Alzheimer disease

The details for the preparation of PLA‐PEG‐Gd were included in the Supporting information. The fabrication of RIF@PLA‐PEG‐Gd/Mal and RIF@PLA‐PEG‐Gd/Mal‐RVG29 is shown in Scheme 1. One hundred milligram each of PLA‐PEG‐Gd and PLA‐PEG‐Mal were dissolved in tetrahydrofuran. One hundred milligram of rifampicin was dissolved in tetrahydrofuran and assisted with ultrasound. The rifampicin solution was added dropwise to the mixed solution of PLA‐PEG‐Gd and PLA‐PEG‐Mal, and ultrasound‐dispersed for 15 min. The above mixture was sonicated for 30 min in an ice bath by using a cell pulverizer (JY92‐IIND, SCIENTZ). During the ultrasonic process, 25 ml of H_2_O was gradually added. The solution was transferred to a dialysis bag (cut‐off MW: 3500 Da) in an ice bath for 24 h. RIF@PLA‐PEG‐Gd/Mal was prepared by freeze‐drying the dialysate. Further, 100 mg of RIF@PLA‐PEG‐Gd/Mal was dissolved in PBS. 10 mg of RVG29 was dissolved in PBS. The concentration of RIF@PLA‐PEG‐Gd/Mal and RVG29 was 0.01 M. Then, the RVG29 solution was added dropwise to the RIF@PLA‐PEG‐Gd/Mal solution under magnetic stirring, followed by a reaction time of 4 h. The solution was dialyzed against a dialysis bag (cut‐off MW: 3500 Da) in an ice bath for 12 h. Finally, RIF@PLA‐PEG‐Gd/Mal‐RVG29 was obtained by freeze‐drying the dialysate.

### 2.3 Characterization

The chemical features of PLA‐PEG‐NH_2_ and PLA‐PEG‐DTPA were carried out by Fourier transform infrared spectroscopy (TENSOR‐27, Bruker, Germany). Nuclear magnetic resonance spectroscopy (NMR, AVANCE III 500 MHz, Bruker, Germany) was used to characterize the modification of DTPA and RVG29 peptides, and the samples were dissolved in deuterated DMSO. Inductively coupled plasma atomic emission spectroscopy (ICP‐AES, Thermo iCAP 7000 SERIES, Thermo, USA) was used to test the content of plutonium in PLA‐PEG‐Gd/Mal and RIF@PLA‐PEG‐Gd/Mal. Dynamic light scattering (DLS, Zeta sizer Nano‐ZS 90, Malvern, USA) was used to characterize the particle size distribution and zeta potential of polymer nanoparticles. Transmission electron microscope (TEM, JEM‐2010F, JEOL, Japan) was used to observe the morphologies and structures of the nanoparticles and synapse structures in the hippocampal CA1 area. In vitro MRI study of PLA‐PEG‐Gd/Mal was scanned on a Gyroscan Intera 1.5 T magnetic resonance imaging (1.5 T MRI, Philips Medical Systems, The Netherlands). The UV–Vis spectrophotometer (UV, V‐3100PC, Mapada, China) was used to evaluate the encapsulation efficiency and loading capacity of rifampicin in nanomedicine. Cell Counting Kit‐8 (CCK‐8) assay was used to evaluate the toxicity of PLA‐PEG‐Gd/Mal, RIF@PLA‐PEG‐Gd/Mal, and RIF@PLA‐PEG‐Gd/Mal‐RVG29 in HT22 cells. Confocal laser scanning microscope (CLSM, LEICA, SP8, Germany) was used to observe the fluorescence state of cells and evaluate the in vitro targeting effect of the RVG29‐targeted drug delivery system.

### 2.4 Animals and drug administration

This study complied with the Chinese Animal Care and Institutional Ethical Guidelines. All experimental procedures strictly followed the regulations of the Institute of Laboratory Animal Science of Jinan University. Male APP_swe_/PS1_dE9_ double transgenic mice (APP/PS1 mice, Tg mice) and female C57BL/6J (Wild type, WT) were purchased from Beijing Huafukang Biotechnology Co., Ltd. Males and females were freely mated for a breeding ratio of 3:1. The mice bred by mating male APP_swe_/PS1_dE9_ double transgenic mice with female C57BL/6J were housed in cages about 3 weeks after birth. APP/PS1 double transgenic mice and C57BL/6J mice were identified by PCR after 1‐month rearing. These breeding mice were kept at the Animal Experiment Center of Jinan University for 12 h light a day. The temperature was 23 ± 2°C and the humidity was 45%–65%. All mice were free to eat and drink. Rifampicin was dissolved in a small amount of DMSO and then diluted to the use concentration with PBS. Other drugs were dissolved in PBS. The effective drug concentrations of RIF, RIF@PLA‐PEG‐Gd/Mal, and RIF@PLA‐PEG‐Gd/Mal‐RVG29 were 20 mg/(kg d). The mice in each group were equally divided into female and male mice. Both WT mice and APP/PS1 mice were injected with the same volume of PBS. All mice were administered via tail vein injection with once every 3 days and a total of eight times. In this test, a total of 32 Tg mice aged 9–10 months were used. Eight WT mice from the same batch were used as the control group. These mice were randomly divided into 5 groups: WT + PBS (*n* = 8), Tg + PBS (*n* = 8), Tg + RIF (*n* = 8), Tg + RIF@PLA‐PEG‐Gd/Mal (*n* = 8), and Tg + RIF@PLA‐PEG‐Gd/Mal‐RVG29 (*n* = 8).

### 2.5 Tissue preparation

Morris's water maze (MWM) experiment was described in the Supporting information. After the MWM experiment, the mice were deeply anesthetized with tribromoethanol, and cardiac perfusion was performed with ice‐cold saline and 4% paraformaldehyde until the effluent became clear and transparent. Then the brain was quickly removed and fixed in 4% paraformaldehyde overnight. The brain was embedded in paraffin for H&E staining and immunofluorescence experiments. To analyze the changes in the synaptic structure of hippocampal CA1 region through transmission electron microscopy, cardiac perfusion was performed with saline until the effluent was clear and transparent. Subsequently, it was fixed by perfusion with 2.5% glutaraldehyde. The CA1 region of each hippocampus was quickly separated and then stored in 2.5% glutaraldehyde at 4°C for further use. For the western blot test, after the mice were bled from the eyeballs, fresh brain tissue was quickly removed on the ice platform, and then stored at −80°C for further use.

### 2.6 Hematoxylin–eosin staining

Hematoxylin–eosin (H&E) staining was used to observe the morphological changes of hippocampal CA1 neurons in mice. The paraffin‐embedded brain tissue was cut into 4 μm sections. These brain tissue sections were dewaxed with xylene and hydrated with ethanol. Then nucleus was stained with hematoxylin, and the cytoplasm was stained with eosin. Tissue sections were sealed and stored between slides and coverslips after dehydration by using ethanol. The pathological changes of neurons in the hippocampal CA1 area were observed under the light microscope.

### 2.7 Immunofluorescence staining

The paraffin‐embedded brain tissue was cut into 4 μm sections. After preheating, these brain tissue sections were then dewaxed in xylene. Then, these brain tissue sections were fixed in ethanol with different concentration gradients (100%, 90%, 80%, 70%, 60%), followed by antigen retrieval. After blocking with 5% bovine serum albumin for 1 h, the sections were incubated with Aβ antibody (1:100) overnight at 4°C. The next day, these sections were washed with PBS and then incubated with the TRITC donkey anti‐rabbit secondary antibody (1:500, Abcam) at room temperature for 1 h. Finally, it was stained with 2‐(4‐amidinophenyl)‐6‐indolecarbamidine dihydrochloride (DAPI). An inverted fluorescence microscope was used to observe the Aβ deposition in the cerebral cortex and hippocampus. The data were analyzed by Image‐pro Plus 6.0.

### 2.8 Western blot analysis

The western blot antibodies used were as follows: PSD95 (1:1000, Cell Signaling Technology), SYP (1:2000, Cell Signaling Technology), and Tubulin (1:5000, Cell Signaling Technology). The blots were scanned using Amersham Imager 600 Ger Imaging System. Results statistics were.

### 2.9 In vivo real‐time imaging

NHS‐Cy5.5 was used to label RIF@PLA‐PEG‐Gd/Mal and RIF@PLA‐PEG‐Gd/Mal‐RVG29 nanoparticles. The mixed solution of Cy5.5 and nanoparticles was allowed to react at room temperature for 4 h in chloroform and dialysis against deionized water for 3 days. The Cy5.5 labeled nanoparticles were finally obtained by lyophilizing. Near‐infrared dye Cy5.5 (excitation wavelength = 675 nm, emission wavelength = 694 nm)‐labeled non‐targeted RIF@PLA‐PEG‐Gd/Mal and brain‐targeted RIF@PLA‐PEG‐Gd/Mal‐RVG29 were injected into C57BL/6J mice (*n* = 3, per group) through the tail vein, respectively. The fluorescent images were taken by IVIS spectrum in vivo imaging system (PerkinEler Instruments [Shanghai] Co., Ltd) at 0.5, 1, 2, 6, 12, 24, 36, 48 h after intravenous injection when mice were anesthetized with tribromoethanol. The brains, hearts, livers, spleens, lungs, and kidneys were removed from mice after the last in vivo fluorescence imaging. The organs were visualized under the in vivo IVIS spectrum imaging system.

### 2.10 In vivo MRI study

All the MRI were recorded after the mice were injected with RIF@PLA‐PEG‐Gd/Mal‐RVG29 (4.8 mg/mL) containing gadolinium contrast agents. Ten‐month‐old C57BL/6J (WT) and APP/PS1 mice were used to test the MRI performance of the drug, respectively. The MRI images were obtained 1 h after the drug injection. For comparison, MRI was performed before the drug injection. In vivo MRI was performed with a 9.4 T horizontal bore scanner. The T1‐weighted spin‐echo (T1SE) sequence parameters were: TR = 300 ms, TE = 10 ms, slice thickness = 1 mm, number of slices = 3256 × 256 matrices, average = 8. Acquisition time with the T1SE sequence was 20 min for one signal average. During MRI experiments, the mice were anesthetized with a mixture of isoflurane (0.75%–1.5%) and carbogen (95% O_2_–5% CO_2_) and their respiratory rate was monitored.

### 2.11 Statistical analysis

The data were first tested for equal variance and normality, and then showed as mean ± SD. Comparisons among multiple groups were made using one‐way ANOVA followed by Bonferroni post hoc pairwise comparisons. Differences were deemed statistically significant where *p* < 0.05. Statistical analysis of the data was performed by using *SPSS19.0* statistical software.

## 3 RESULTS AND DISCUSSION

### 3.1 Characteristics of nanoparticles

**FIGURE 1:**
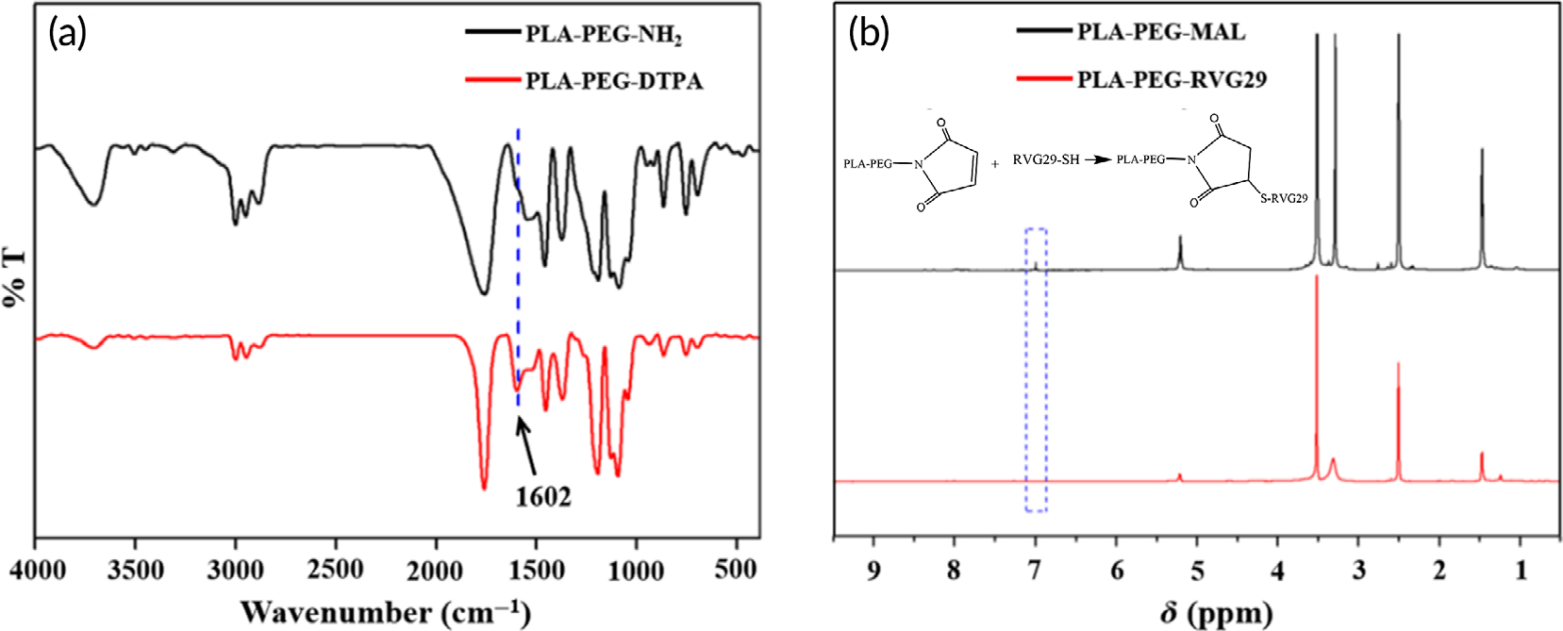
(a) The FT‐IR spectra of PLA‐PEG‐NH_2_ and PLA‐PEG‐DTPA. (b) ^1^H NMR result of PLA‐PEG‐MAL and PLA‐PEG‐RVG29

The FT‐IR spectra of PLA‐PEG‐NH_2_ and PLA‐PEG‐DTPA are shown in Figure 1a. Compared to PLA‐PEG‐NH_2_, a new peak appeared at 1602 cm^−1^ on PLA‐PEG‐DTPA. This was the stretching vibration of C=O on the newly formed amide I band of the amide bond, indicating that DTPA and the aminated copolymer reacted and connected by amide bonds. ^1^H NMR result further confirmed that PLA‐PEG‐DTPA was successfully prepared (Figure S1). PLA‐PEG‐DTPA: ^1^H NMR [DMSO‐d6], *δ* [ppm]: 5.21 (PLA, C—H), 3.50 (PEG, —CH_2_), 3.02, 2.92, 2.86 (DTPA, —CH_2_), 1.98 (DTPA, —CH_2_—CH_2_) 1.23, 1.47 (PLA, —CH_3_). As shown in Figure 1b, overlapping peaks of residual water and RVG29 amino acid of PLA‐PEG‐RVG29 appeared in 3.2–3.4 ppm. The characteristic peak of the maleimide group (6.9 ppm) did not show in PLA‐PEG‐RVG29 compared with PLA‐PEG‐MAL, indicating that the maleimide group had reacted with the thiol group on the RVG29. This further confirms that the RVG29 polypeptide was successfully modified on the copolymer to prepare the targeted PLA‐PEG‐RVG29 nanoparticles.

**FIGURE 2:**
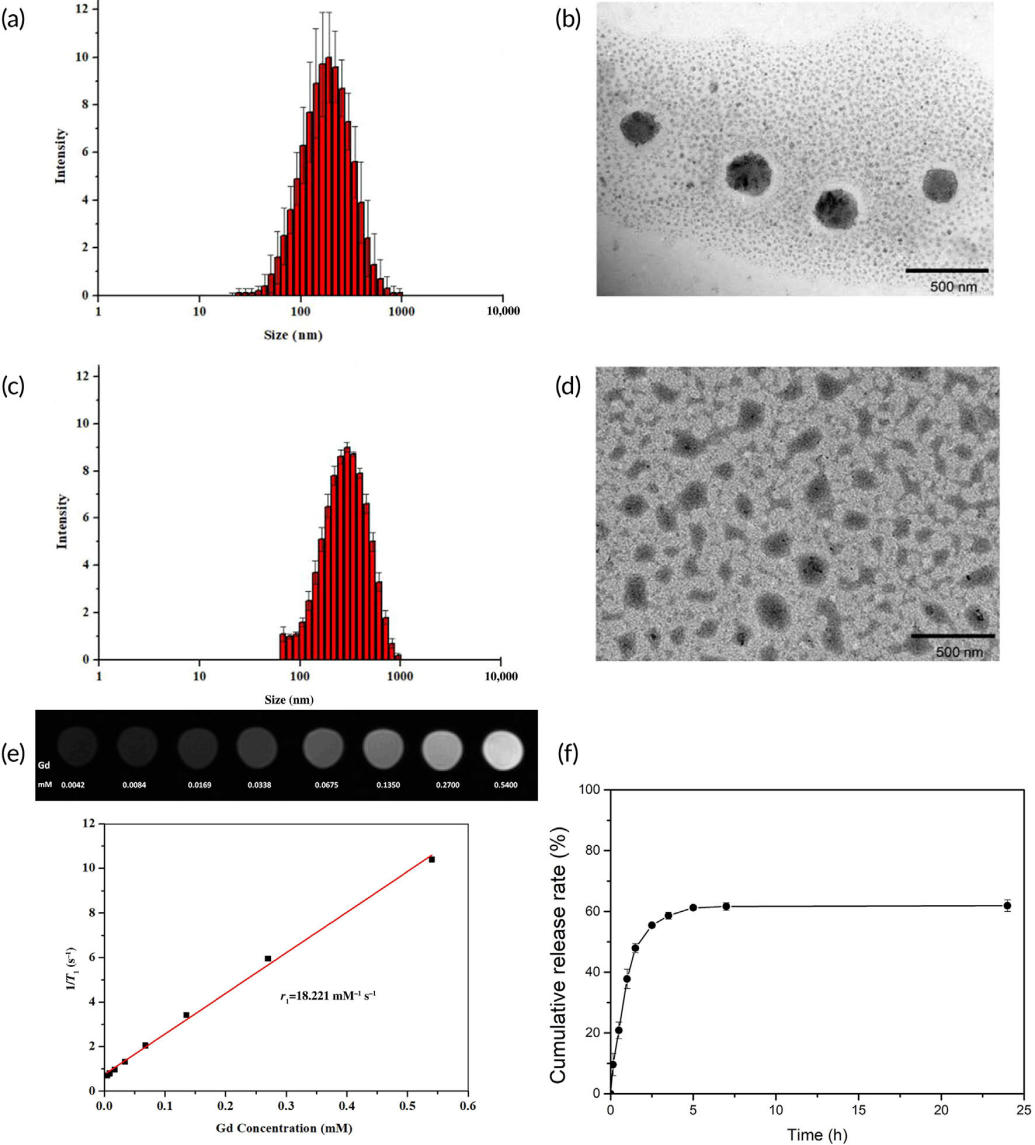
(a) Size distribution of PLA‐PEG‐Gd/Mal. (b) Size distribution of RIF@PLA‐PEG‐Gd/Mal‐RVG29. (c) TEM image of PLA‐PEG‐Gd/Mal. (d) TEM image of RIF@PLA‐PEG‐Gd/Mal‐RVG29. (e) MRI of PLA‐PEG‐Gd/Mal with different concentrations in water. (f) A plot of *T*_1_ relaxation rate (1/*T*_1_) against Gd concentration, corresponding to (e). (g) Release profiles of rifampicin from RIF@PLA‐PEG‐Gd/Mal‐RVG29 micelles

As shown in Figure 2a,c, the average particle diameter of PLA‐PEG‐Gd/Mal was 176.6 nm, and the Zeta potential was +8.37 mV. The blank carrier exhibited a uniform spherical shape. Besides, the average particle diameter of the drug‐loaded RIF@PLA‐PEG‐Gd/Mal‐RVG29 reached 263.1 nm, and the Zeta potential was −9.49 mV (Figure 2b,d). According to the results of ICP‐AES, the mass of Gd in 1 mg of PLA‐PEG‐Gd/Mal was 0.0283 mg and the loading factor of Gd in PLA‐PEG‐Gd/Mal was 2.51% (Table 1). The nano‐drug carrier was composed of hydrophobic PLA chains and hydrophilic PEG chains, and the hydrophobic PLA was easy to combine with hydrophobic rifampicin, which was beneficial for the prepared nanoparticles to encapsulate more drugs. There was almost no difference in loading capacity and encapsulation efficiency of rifampicin in PLA‐PEG‐Gd/Mal and PLA‐PEG‐Gd/Mal‐RVG29 (Figure S2 and Table 2), indicating the modification of RVG29 did not influence the formation of the micelle, which remains its excellent drug carry ability. The loading capacity of rifampicin in PLA‐PEG‐Gd/Mal‐RVG29 reached 16.4% and encapsulation efficiency was 49.2%. The loading capacities of most nanomaterials were less than 10% in other researches.^36^, ^37^ Notably, our study discovered a new material that increases the loading capacity to 16.7% and 16.4%. As shown in Figure 2e, the MRI image gradually became brighter as the concentration of Gd increased. Besides, the slope of the straight line obtained by fitting the data points was the T1 relaxation efficiency and the curve fits well (Figure 2f). This indicated that the sample had excellent MRI imaging performance. The release properties of rifampicin from RIF@PLA‐PEG‐Gd/Mal‐RVG29 were shown in Figure 2g, shown that the cumulative release of rifampicin reached a peak after 7 h, and the cumulative release reached 61%.

**TABLE 1:** ICP‐AES result of PLA‐PEG‐Gd/Mal and RIF@PLA‐PEG‐Gd/Mal

Sample	Concentration average	Concentration RSD
PLA‐PEG‐Gd/Mal	7.44 ppm	2.51%

**TABLE 2:** Drug‐loaded rate and encapsulation rate of RIF@PLA‐PEG‐Gd/Mal and RIF@PLA‐PEG‐Gd‐RVG29

Sample	Loading capacity	Encapsulation efficiency
RIF@PLA‐PEG‐Gd/Mal	16.7%	50%
RIF@PLA‐PEG‐Gd/Mal‐RVG29	16.4%	49.2%

### 3.2 In vitro cellular uptake and cytotoxicity studies of nanoparticles

**FIGURE 3:**
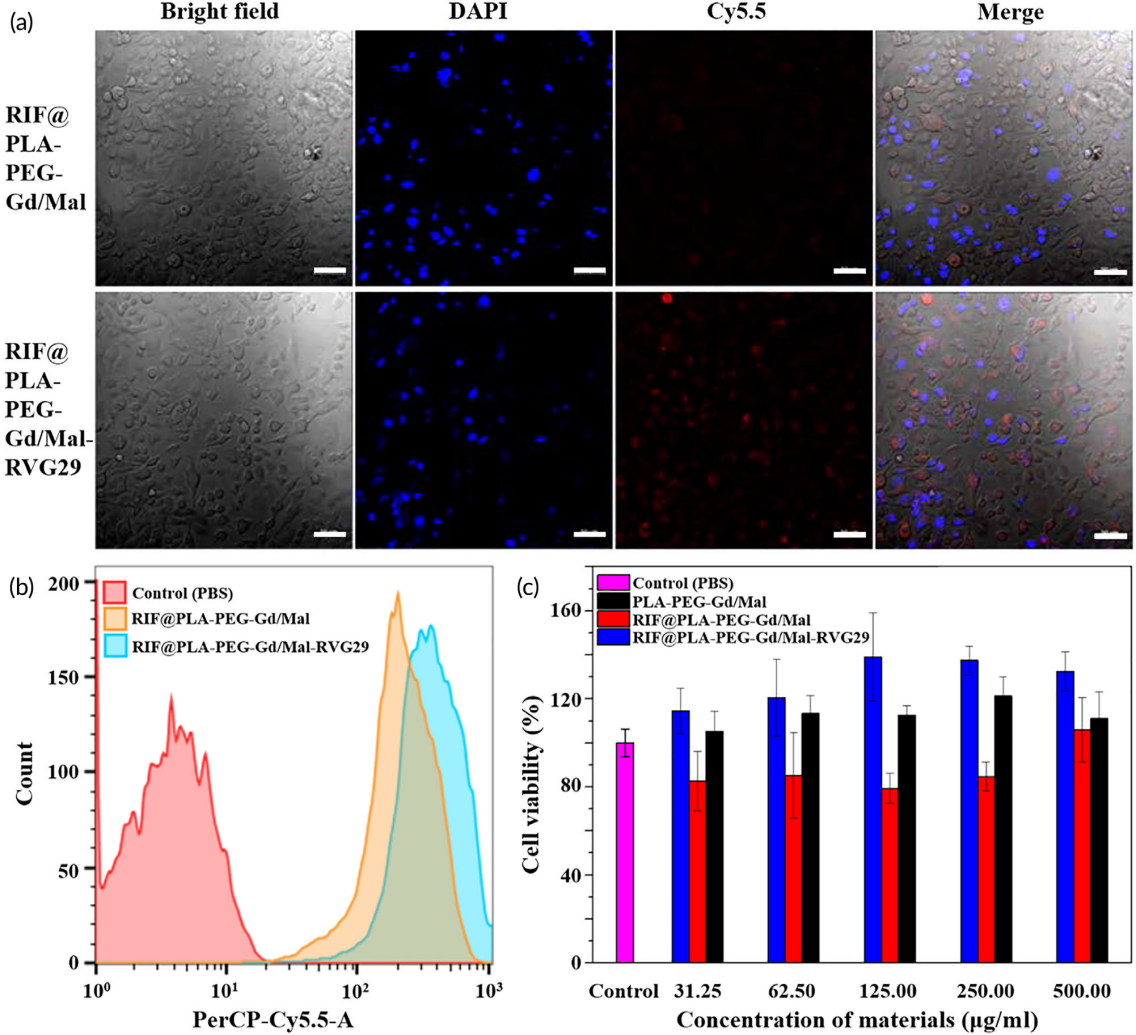
(a) Confocal images of Cy5.5‐labeled RIF@PLA‐PEG‐Gd/Mal (non‐targeted) and RIF@PLA‐PEG‐Gd/Mal‐RVG29 (targeted). Scale bars: 50 μm. (b) Quantitative analysis of the Cy5.5 fluorescence density in bEnd.3 cells. (c) CCK‐8 results of PLA‐PEG‐Gd/Mal, RIF@PLA‐PEG‐Gd/Mal and RIF@PLA‐PEG‐Gd/Mal‐RVG29 tested by using HT22 cells with different concentrations. Data are presented as mean ± SD (*n* = 3); *p < 0.05; **p < 0.01

The immortalized mouse brain capillary endothelial cell line (bEnd.3) is often used as an in vitro BBB model for drug uptake and transport studies.^38^ Most transport across the BBB occurs in a selective and tightly regulated manner and is controlled by carrier‐mediated transport and receptor‐mediated transport (RMT).^39^ nAChRs are expressed on the BBB, astrocytes, neurons, and peripheral tissues.^40^ RVG29 specifically binds to nAChRs for achieving BBB transport through RMT.^31^, ^41^ Therefore, we examined whether RVG29 could increase the uptake of NPs by bEnd.3 cells. When bEnd.3 cells were co‐cultured with non‐targeted RIF@PLA‐PEG‐Gd/Mal NPs and targeted RIF@PLA‐PEG‐Gd/Mal‐RVG29 NPs for 4 h, the uptake of NPs by bEnd.3 cells was observed by CLSM. Red fluorescence was the characteristic fluorescence of the fluorescent dye Cy5.5, and blue fluorescence was the characteristic fluorescence of DAPI. Through binding to the cell surface and undergoing endocytosis, arginine‐rich peptides including RVG29 have been demonstrated to achieve the ability to overcome endocytic vesicles. They have been detected in the cytosol, which is mainly attributed to the promotion of endosomal escape.^42^ Some groups have argued that arginine‐rich peptides will destabilize or disrupt the endosomal membrane and subsequently access the cytosol. While others claim that escape from endosomes occurs through membrane fusion in which the guanidinium groups play a major role.^43^ However, recent studies on RVG29‐coupled liposomes proposed that the endosomal escape is mediated by lipid fusion to the endosome membrane followed by membrane leakage. NPs were distributed in the cytoplasm and nucleus, and the red fluorescence intensity in the cells of the targeted group was significantly stronger than that of the non‐targeted group (Figure 3a). Flow cytometry was used to compare the uptake of targeted and non‐targeted NPs by in vitro cells. The fluorescence intensity of bEnd.3 cells could be effectively increased in the NPs equipped with RVG29, indicating that RIF@PLA‐PEG‐Gd/Mal‐RVG29 could specifically target to the bEnd.3 cells. What is more, Liu et al. reported an in vitro BBB model, PAMAM‐PEG‐RVG29/DNA NPs, which have a higher crossing efficiency of BBB than PAMAM/DNA NPs. The reported gene expression of the PAMAM‐PEG‐RVG29/DNA NPs observed in the brain was significantly higher than unmodified NPs.^31^ The above results indicate that RVG29 modified NPs showed great potential to be applied in designing brain‐targeted drug delivery systems. The toxicity evaluation of NPs to HT22 cells is shown in Figure 3c. The cell viability in PLA‐PEG‐Gd/Mal, RIF@PLA‐PEG‐Gd/Mal, and RIF@PLA‐PEG‐Gd/Mal‐RVG29 treated at various concentrations are above 80%, indicating the NPs did not cause cytotoxicity to HT22 cells.

### 3.3 In vivo imaging evaluation of near‐infrared dye Cy5.5‐labeled RIF@PLA‐PEG‐Gd/Mal and RIF@PLA‐PEG‐Gd/Mal‐RVG29

**FIGURE 4:**
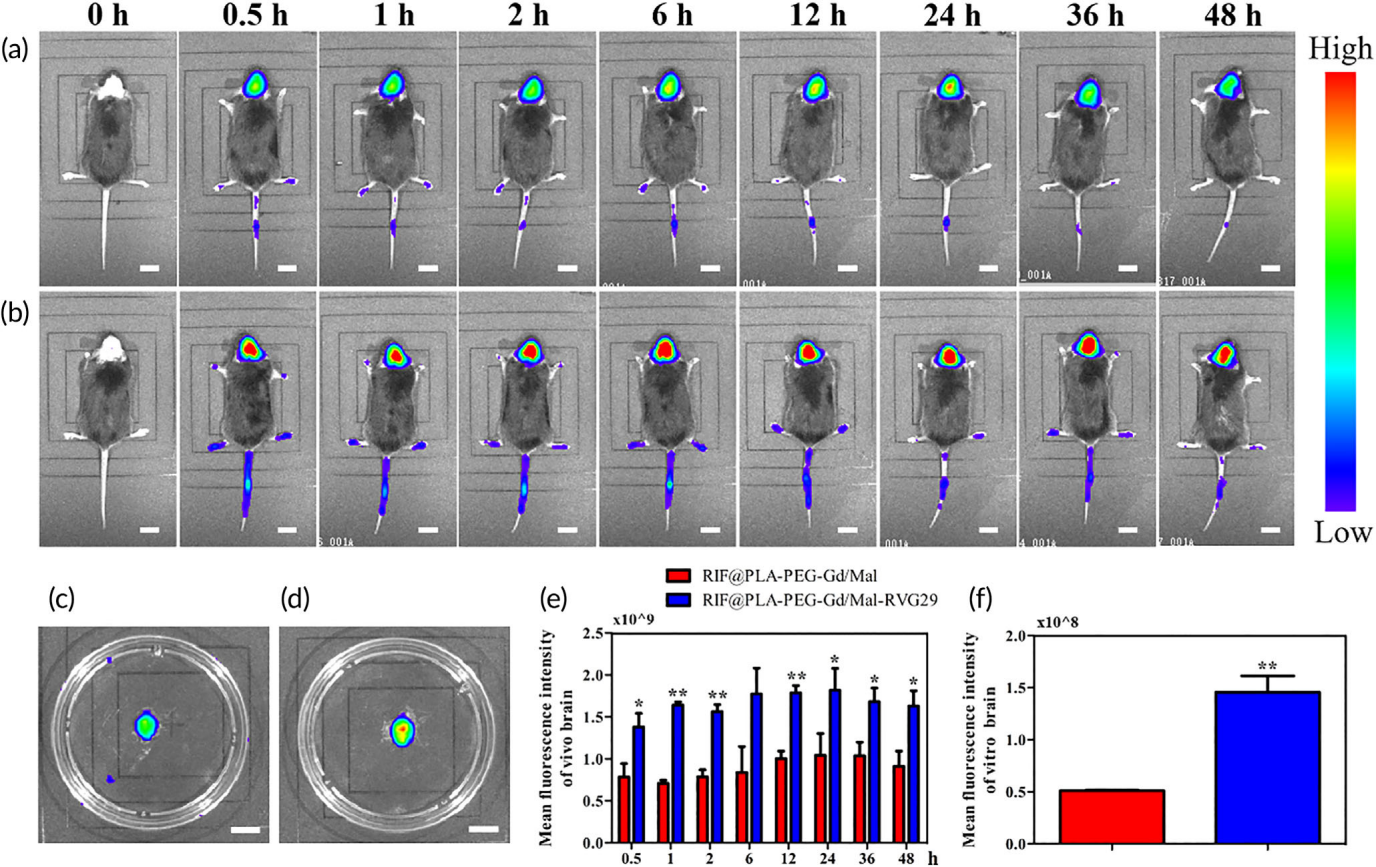
In vivo distribution of Cy5.5‐labeled non‐targeted (RIF@PLA‐PEG‐Gd/Mal) NPs and RVG29‐targeted (RIF@PLA‐PEG‐Gd/Mal‐RVG29) NPs after intravenous administration. (a) Fluorescence images of mice injected with RIF@PLA‐PEG‐Gd/Mal NPs. (b) Fluorescence images of mice injected with RIF@PLA‐PEG‐Gd/Mal‐RVG29 NPs. (c) Fluorescence images of ex vivo brain injected with RIF@PLA‐PEG‐Gd/Mal NPs. (d) Fluorescence images of ex vivo brain injected with RIF@PLA‐PEG‐Gd/Mal‐RVG29 NPs. Images of ex vivo brain were taken at 48 h after injection. (e) Mean fluorescence intensity for in vivo brain in the Cy5.5 channel. (f) Mean fluorescence intensity for ex vivo brain in the Cy5.5 channel. Mean fluorescence intensity was quantified by using Living Image version. Data are presented as mean ± SD (*n* = 3); *p < 0.05; **p < 0.01. Scale bars: 1 cm

In this paper, the amount of Cy5.5‐NHS addition was calculated according to the ratio of the amount of RIF@PLA‐PEG‐Gd/Ma, and the amount of Cy5.5‐NHS added in RIF@PLA‐PEG‐Gd/Mal‐RVG29 was consistent. The hair of mice affects the results of fluorescence imaging. In this study, only the head hair of mice was removed for in vivo fluorescence imaging, but we did not study the distribution of NPs in other organs. Figure 4a,b shows the in vivo fluorescence images at 0.5 , 1 , 2 , 6 , 12 , 24 , 36 , and 48 h after the tail vein injection of RIF@PLA‐PEG‐Gd/Mal and RIF@PLA‐PEG‐Gd/Mal‐RVG29. Figure 4c,d shows the fluorescence image of the excised brain after 48 h injection. The in vivo fluorescence intensity of brain‐targeted RIF@PLA‐PEG‐Gd/Mal‐RVG29 in the brain was significantly higher than non‐targeted RIF@PLA‐PEG‐Gd/Mal (Figure 4a,b), similar to the results found ex vivo brain (Figure 4c,d). Targeted RIF@PLA‐PEG‐Gd/Mal‐RVG29 nanoparticles began to accumulate significantly in the brain within 30 min and concentrated in the brain for at least 48 h, while non‐targeted RIF@PLA‐PEG‐Gd/Mal nanoparticles were less accumulated in the brain. The quantitative fluorescence intensity in the targeted RIF@PLA‐PEG‐Gd/Mal‐RVG29 treated group was higher than that in the non‐targeted RIF@PLA‐PEG‐Gd/Mal treated group both in vitro and in vivo. The fluorescence intensity of RIF@PLA‐PEG‐Gd/Mal‐RVG29 in the harvested brain tissue was almost three‐fold higher than the on‐targeted RIF@PLA‐PEG‐Gd/Mal group. These results demonstrate that RIF@PLA‐PEG‐Gd/Mal‐RVG29 has good brain‐targeted performance in vivo. It is a pity that it should be the nanoparticle distribution in the brain tissue should also be confirmed from the brain tissue section using Cy5.5‐labeled nanoparticles in this study.

### 3.4 MRI of amyloid plaque detection in mice

**FIGURE 5:**
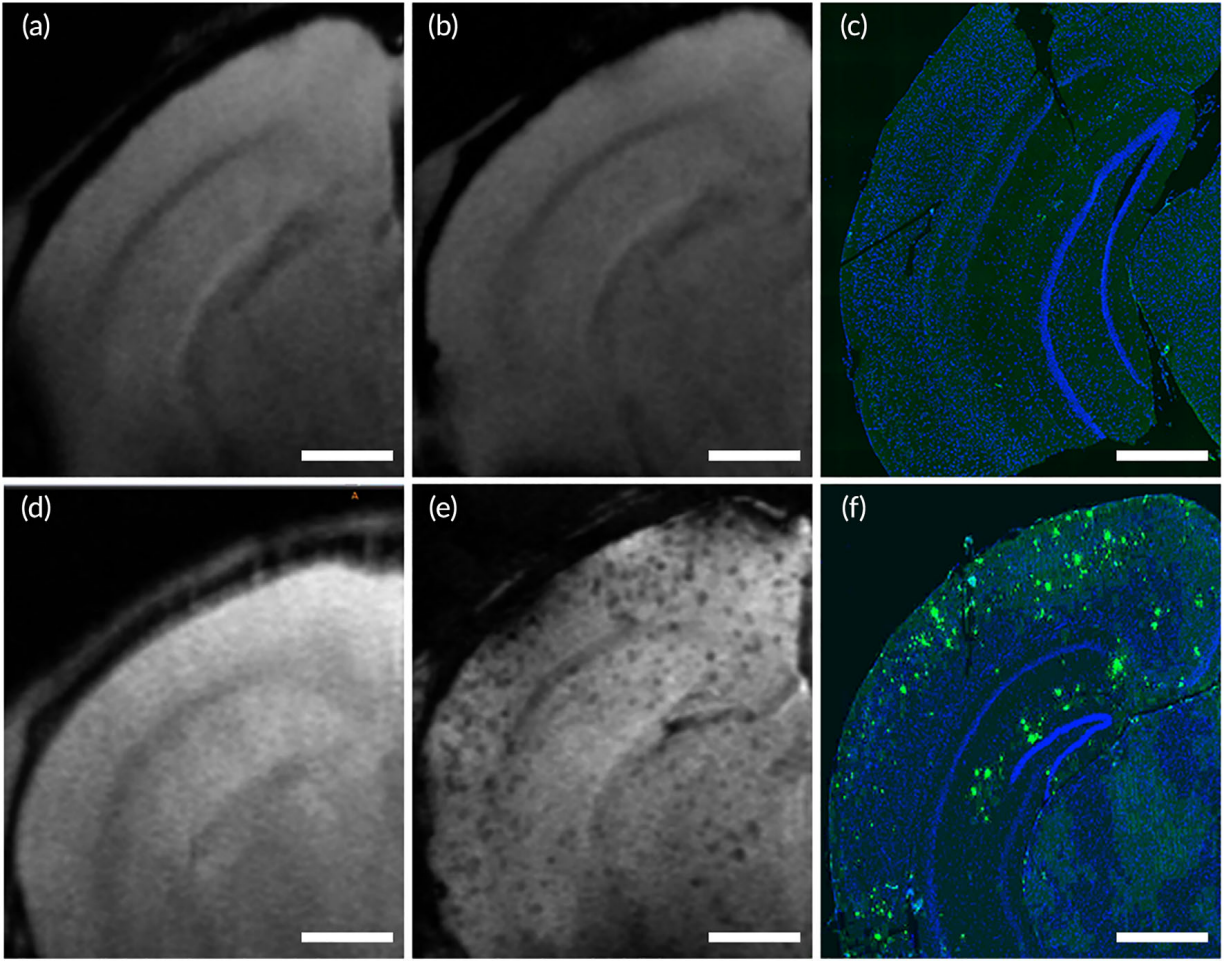
Comparison between detection of amyloid plaques by Gd‐assisted MRI and immunofluorescence staining of histological sections in WT and APP/PS1 mice. (a) MRI of WT mice before injection. (b) Gd‐assisted MRI of WT mice at 1 h of injection. (c) The immunofluorescence staining of histological sections in WT mice. (d) MRI of APP/PS1 mice before injection. (e) Gd‐assisted MRI of APP/PS1 mice at 1 h of injection. (f) The immunofluorescence staining of histological sections in APP/PS1 mice. Both WT and APP/PS1 mice were injected with RIF@PLA‐PEG‐Gd/Mal‐RVG29 containing Gd contrast agent by intravenous injection. Scale bars: 500 μm

Gadolinium (Gd) chelates are the most widely used MRI contrast agents. Gd can be used as an MRI contrast agent to detect amyloid plaques. By adjusting the magnetic resonance signal, Gd is able to shorten the T1 relaxation time of surrounding protons and increase the contrast between amyloid plaques and brain parenchyma.^44^ The hydrophilic Gd contrast agent can increase the signal of the tissue around the amyloid plaque and make the amyloid plaque appear black spots.^45^, ^46^ In order to confirm the diagnostic properties of the nanoparticles for AD, MRI with Gd‐assisted imaging was performed on 10‐month‐old WT and APP/PS1 mice, respectively. Immunofluorescence staining showed that amyloid protein was not detected in WT mice (Figure 5c), but was detected in the hippocampus and cortex of APP/PS1 mice. Before contrast agent injection, hypointense spots were not visible in the MRI of WT and APP/PS1 mice (Figure 5a,d). After contrast agent injection, the hypointense spots could be seen in the cortex and hippocampus of APP/PS1 mice (Figure 5e), but they did not show in WT mice (Figure 5b). Immunofluorescence staining of Aβ in brain tissue further confirmed that the hypointense spots on magnetic resonance images were amyloid plaques (Figure 5f). The above results support that nanoparticles have the ability to diagnose amyloid plaques in vivo. Therefore, the prepared nanomedicine has the potential in the diagnosis of AD and evaluation of drug efficacy through detecting amyloid plaques in the brain by MRI. Distinct from the reported materials, such as the PAMAM‐PEG‐RVG29/DNA,^31^ we designed and fabricated the RIF@PLA‐PEG‐Gd/Mal‐RVG29, which offers the advantages of biocompatibility, brain targeting, the controllable release of carried drugs, and the ability of disease diagnosis. However, the toxicity of NPs needs to be concerned. Gd is a toxic MRI agent. The leaking of Gd from the NPs will be studied in our future studies.

### 3.5 RIF@PLA‐PEG‐Gd/Mal‐RVG29 improved the spatial learning and memory capability of APP/PS1 mice in the MWM

**FIGURE 6:**
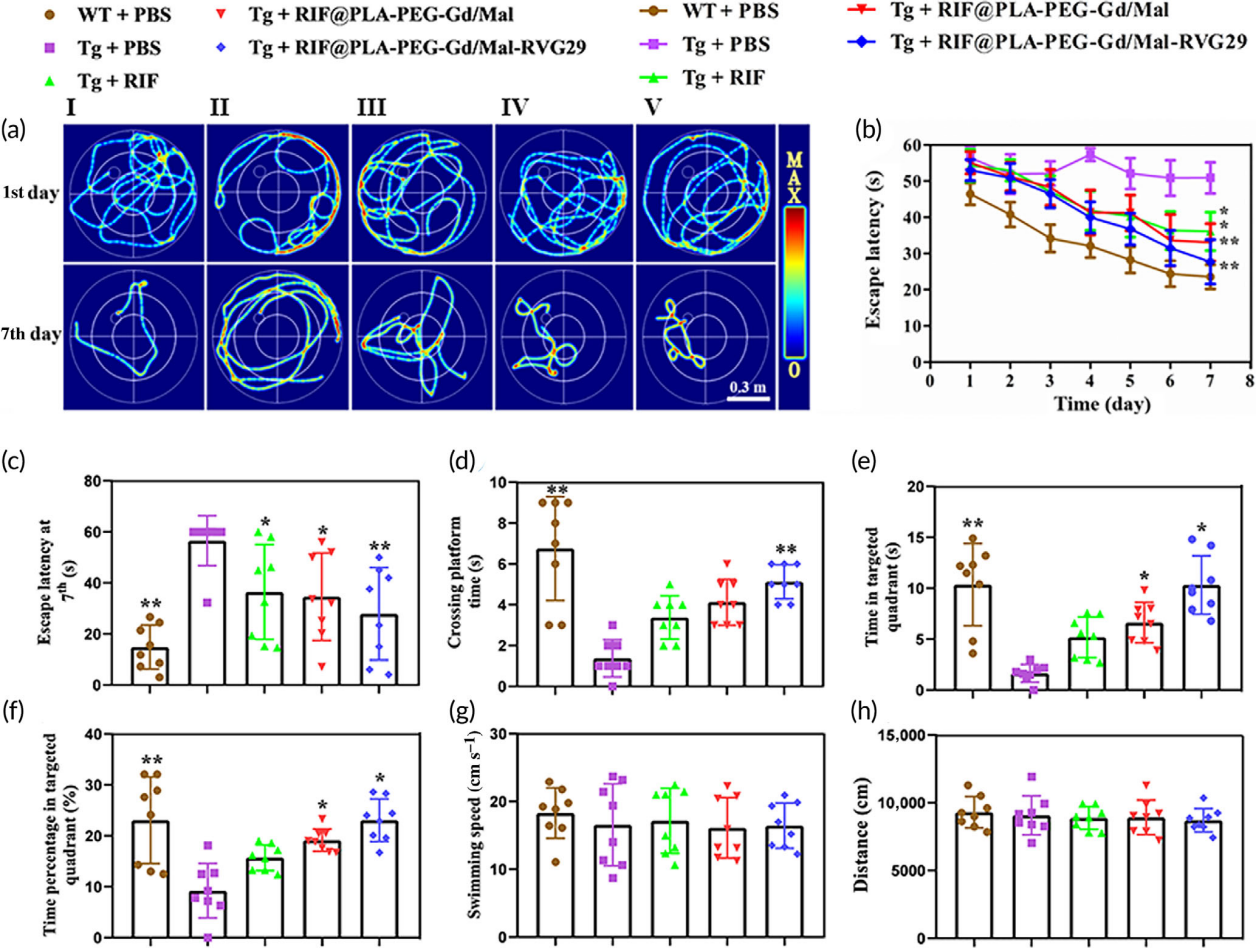
The effect of RIF@PLA‐PEG‐Gd/Mal‐RVG29 on cognitive function of APP/PS1 mice. (a) The trajectories of mice on the first and the seventh days. (I) WT + PBS, (II) Tg + PBS, (III) Tg + RIF, (IV) Tg + RIF@PLA‐PEG‐Gd/Mal, (V) Tg + RIF@PLA‐PEG‐Gd/Mal‐RVG29. (b) Latency time of mice in consecutive 7 days. (c) Comparison of escape latency of each group at Day 7. (d) The times of crossing the platform in the spatial probe trial. (e) Time spent in the targeted quadrant. (f) Proportion of time spent in the targeted quadrant. (g) The swimming speed of the mice on the first day. (h) The total swimming distance of mice on the first day. *n* = 8 per group. Values are presented as mean ± SD. *p < 0.05; **p < 0.01 compared to Tg + PBS group

In this study, APP_swe_/PS1_dE9_ transgenic mice were selected as the ideal animal model for AD study. A large amount of Aβ deposits began to appear at the age of 4–6 months, and gradually increased with age. Memory and cognitive disorders appeared at the age of 10–12 months.^47^ Therefore, 9–10‐month‐old APP/PS1 mice were selected as the study object. To study the effect of RIF@PLA‐PEG‐Gd/Mal‐RVG29 on the learning and memory abilities of APP/PS1 double transgenic mice, a Morris water maze test was performed. The directional navigation experiment was carried out for 7 days. The trajectories of mice are shown in Figure 6a. As shown in Figure 6b, the escape latency of APP/PS1 mice (Tg + PBS group) was observably increased, which was significantly different from age‐matched wild‐type mice (WT + PBS group) (*p* < 0.01). Compared with Tg mice treated with PBS, the escape latency of Tg mice treated with RIF, RIF@PLA‐PEG‐Gd/Mal, and RIF@PLA‐PEG‐Gd/Mal‐RVG29 were shortened. Among all drug treatment groups, the escape latency of Tg mice treated with RIF@PLA‐PEG‐Gd/Mal‐RVG29 showed an observable decrease (on the seventh day, *p* < 0.01), indicating that RIF@PLA‐PEG‐Gd/Mal‐RVG29 effectively improve the abilities of spatial memory and learning memory of Tg mice.

After the directional navigation experiment, the memory ability of the mice was detected by a space exploration experiment. The frequency of crossing the platform, and the time of spending in the quadrant of the platform are generally used to detect the ability of memory retrieval, reproduction, and storage of animals. As shown in Figure 6c–f, for the Tg + PBS group, the frequency of crossing the platform was significantly less than in other groups, as well as the time and proportion of staying in the quadrant of the platform were obviously shortened. By contrast, Tg mice treated with RIF@PLA‐PEG‐Gd/Mal and RIF@PLA‐PEG‐Gd/Mal‐RVG29 were spent more time in the targeted quadrant and the frequency of crossing the platform area. However, for the natural rifampicin treatment group, the memory ability of Tg mice was improved, but there was no statistical significance (*p* > 0.05). There was no significant difference in swimming speed and total swimming distance among all groups (on first day, *p* > 0.05), indicating that the genotype of the mouse and exercise ability did not have an effect on the tests of cognitive function (Figure 6g,h). Thus, RIF@PLA‐PEG‐Gd/Mal and RIF@PLA‐PEG‐Gd/Mal‐RVG29 were beneficial for improving the spatial learning and memory abilities in APP/PS1 mice. It might be that nanomaterials improve the efficacy of rifampin. Because of the reducing frequency of natural rifampicin usage and rapid clearance of the drug, the plasma concentration might not have reached the effective treatment concentration. Therefore, the therapeutic effect of natural rifampicin on APP/PS1 mice was not as good as the rifampicin‐loaded nanoparticles.

### 3.6 RIF@PLA‐PEG‐Mal/Gd‐RVG29 reduced Aβ protein in hippocampus and cortex

**FIGURE 7:**
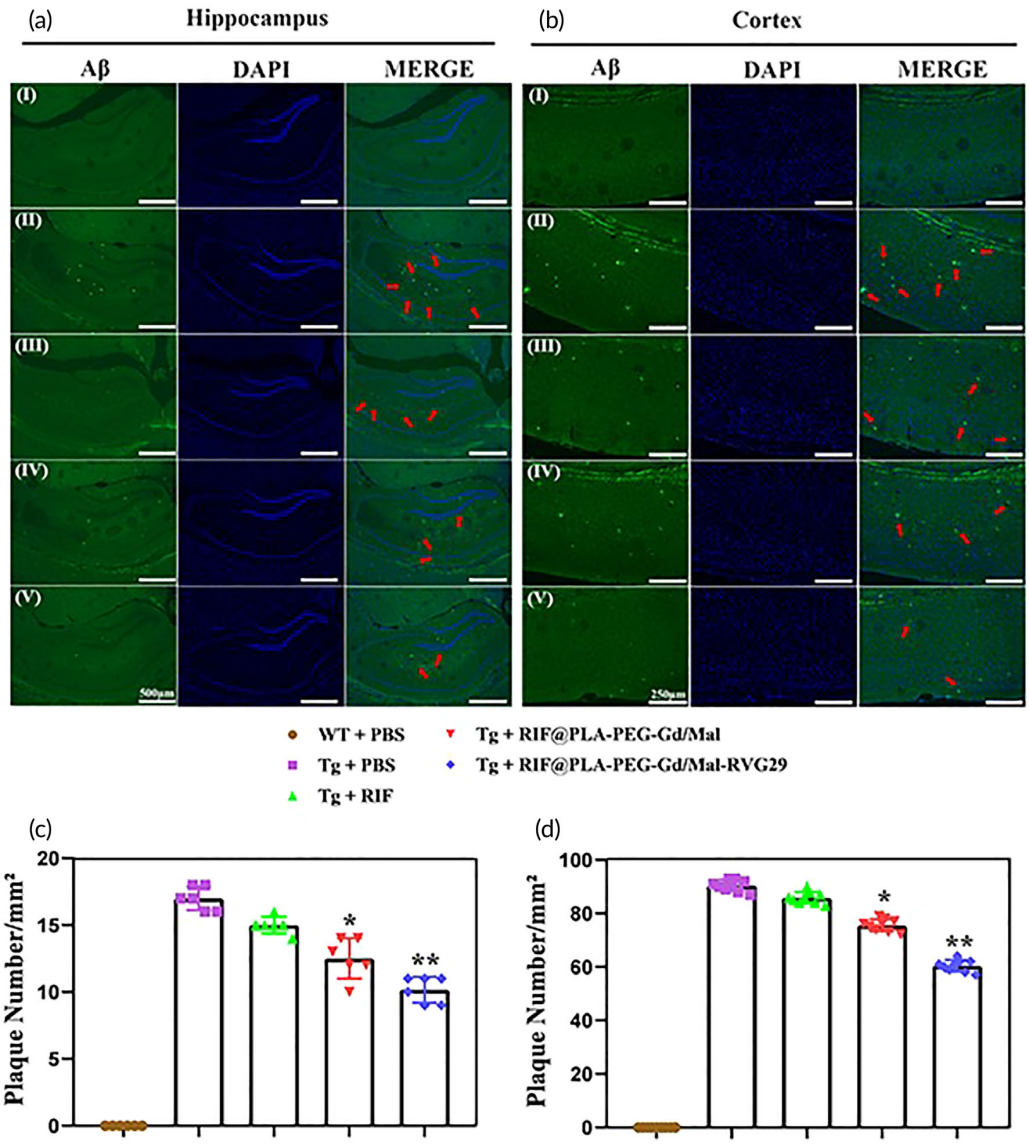
RIF@PLA‐PEG‐Gd/Mal‐RVG29 reduces Aβ deposition in hippocampus. (a) Aβ deposition in the hippocampus. Scale bars: 500 μm (Magnification x5). (b) Aβ deposition in the cortex. Strong green fluorescence signal is Aβ deposition. Scale bars: 250 μm (magnification: ×10). (I) WT + PBS, (II) Tg + PBS, (III) Tg + RIF, (IV) Tg + RIF@PLA‐PEG‐Gd/Mal, (V) Tg + RIF@PLA‐PEG‐Gd/Mal‐RVG29. *n* = 3 per group. Values are presented as mean ± SD. *p < 0.05; **p < 0.01 compared to Tg + PBS group

Three important areas of spatial memory function and the transition from short‐term memory to long‐term memory are the hippocampus, entorhinal cortex, and cingulate cortex. The damage to these areas is related to the memory loss of AD.^48^ The pathogenesis of AD is complex. The accumulation and deposition of Aβ play a key role in the pathogenesis of AD.^49^ The formation and deposition of Aβ in the cortex and hippocampus are directly related to learning and memory deficits in AD.^50^ Previous studies reported that rifampicin has the ability to enhance Aβ clearance.^13^ As shown in Figure 7, we used immunofluorescence to detect the expression levels of Aβ in the hippocampus and cortex. A large amount of Aβ deposition was observed in the hippocampus and cortex of APP/PS1 mice, while Aβ deposition was not observed in WT mice. Aβ protein is scattered and irregular in the hippocampus (Figure 7a) and cortex (Figure 7b) in 9–10 month‐old Tg mice. After treatment with RIF, RIF@PLA‐PEG‐Gd/Mal, RIF@PLA‐PEG‐Gd/Mal‐RVG29, Aβ deposition in the hippocampus and cortex showed a decreasing trend. Brain‐targeted RIF@PLA‐PEG‐Gd/Mal‐RVG29 showed a more obvious reduction in Aβ protein content (Figure 7a(I, II, V),b(I, II, V)). This result indicated that RVG29‐modified nanoparticles loaded with rifampicin was a promising approach to reducing Aβ deposition. In our study, we showed the levels of Aβ in the brain of these mice by Immunofluorescence staining, but ELISA or Western blot will be included for quantitative comparison in our future studies.

### 3.7 Effect of RIF@PLA‐PEG‐Gd/Mal‐RVG29 on synaptic structure and synapse protein

**FIGURE 8:**
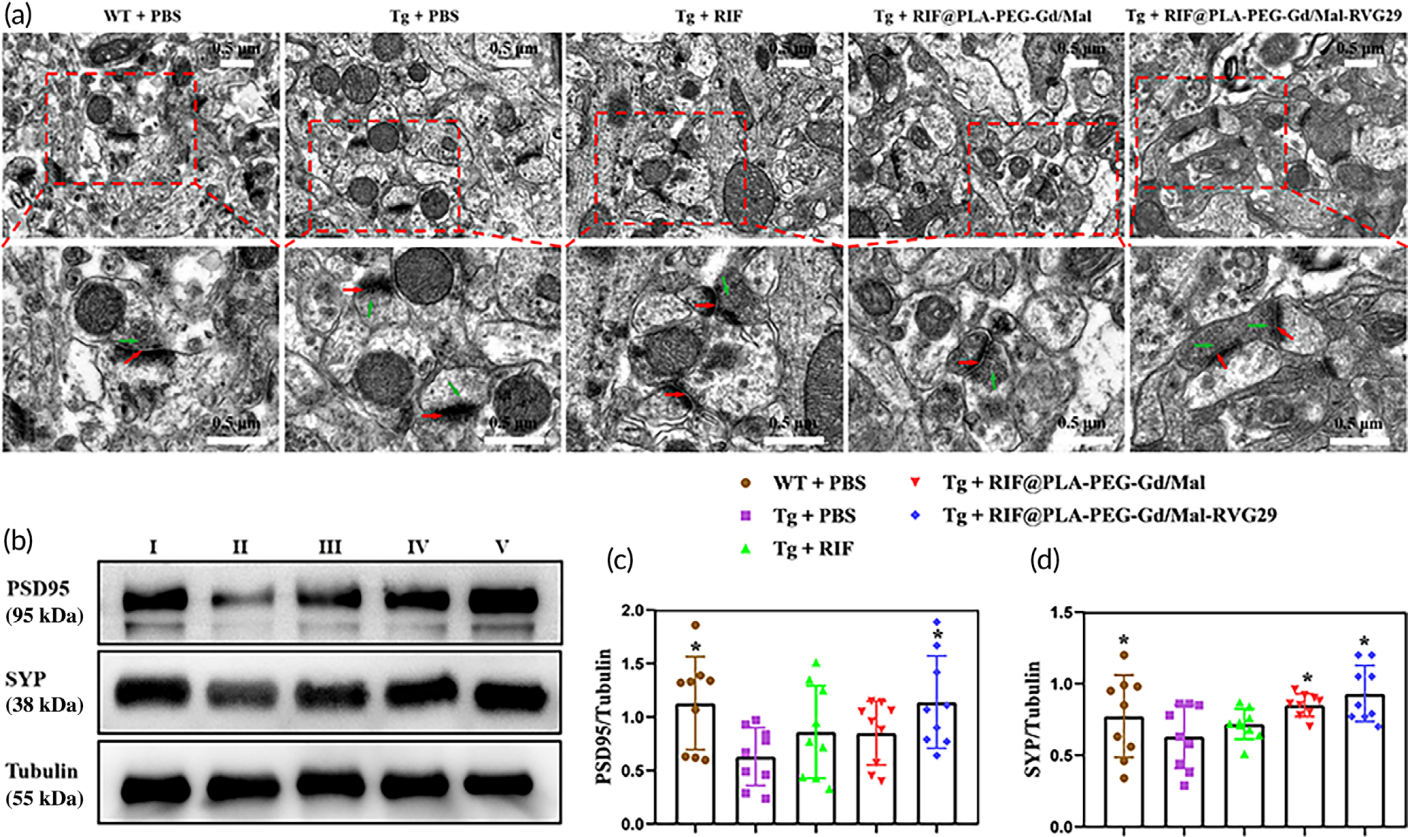
The effect of RIF@PLA‐PEG‐Gd/Mal‐RVG29 on synaptic structure and synapse protein in APP/PS1 mice. (a) The ultrastructure in CA1 region of the hippocampus. Red arrows: synaptic cleft, green arrows: synaptic vesicles. Scale bars: 0.5 μm. (b) Western blotting for detecting PSD95 and SYP expression in the brain of the mice. (I) WT + PBS, (II) Tg + PBS, (III) Tg + RIF, (IV) Tg + RIF@PLA‐PEG‐Gd/Mal, (V) Tg + RIF@PLA‐PEG‐Gd/Mal‐RVG29. Quantitative analysis of the blots showed levels of PSD95 (c) and SYP (d). *n* = 3 per group. Data are described as mean ± SD, *p < 0.05 compared to Tg + PBS group

Synaptic plasticity is the basis of learning and memory.^51^ The deposition of Aβ leads to the destruction of synaptic signaling pathways and dendritic spines, thereby affecting the morphology and function of synapses and leading to memory and behavior defects.^52^ The production of Aβ at the dendrites and axons will partly reduce the number and plasticity of synapses.^53^ Postsynaptic density protein 95 (PSD95) and synaptophysin (SYP) is closely related to synaptic function proteins. PSD95 plays an important role in the plasticity, stability of synapses, and the repair of peripheral nerves after injury.^54^ As a presynaptic plasticity‐related protein, SYP expression indirectly reflects the number, distribution, and density of synapses.^55^ Therefore, the effect of RIF@PLA‐PEG‐Gd/Mal‐RVG29 on synaptic ultrastructure and synaptic protein PSD95 and SYP in Tg mice was studied. As shown in Figure 8, RIF@PLA‐PEG‐Gd/Mal‐RVG29 could promote the repair of damaged synaptic structures in the hippocampal CA1 region and increase the expression of synaptic‐related proteins (PSD95, SYP). As shown in Figure 8a, the three layers of the presynaptic membrane, intersynaptic space, and post‐synaptic membrane in the synaptic structure of hippocampal CA1 area of WT mice are clearly visible under TEM. The presynaptic membrane contains many clear synaptic vesicles. However, the synaptic structure of Tg mice treated with PBS has been damaged. The structure of the presynaptic membrane, synaptic cleft, and the postsynaptic membrane is unclear. The number of synaptic vesicles was reduced and there were few clear synaptic vesicles. RIF, RIF@PLA‐PEG‐Gd/Mal, and RIF@PLA‐PEG‐Gd/Mal‐RVG29 treatments repaired the synapse structure and increased the number of clear synaptic vesicles.

TEM results showed that the changes in synapse structure of mice treated with these three drugs did not have an obvious difference. Western blot was used to detect two synaptic proteins (PSD95, SYP) in mouse brain tissue to reveal the structure of synapses (Figure 8b). Compared with the WT + PBS group, the expression levels of PSD95 and SYP in the Tg + PBS group were obviously reduced. The expression levels of PSD95 and SYP in Tg + RIF and Tg + RIF@PLA‐PEG‐Gd/Mal groups increased, and the expression levels of PSD95 and SYP in the Tg + RIF@PLA‐PEG‐Gd/Mal‐RVG29 group increased obviously (Figure 8c,d). These results indicated that RIF@PLA‐PEG‐Gd/Mal‐RVG29 treatment of Tg mice effectively enhanced expression levels of PSD95 and SYP, and reestablished synaptic ultrastructure.

### 3.8 Effect of RIF@PLA‐PEG‐Gd/Mal‐RVG29 on the pathomorphology of hippocampal and cortex neurons in Tg mice

**FIGURE 9:**
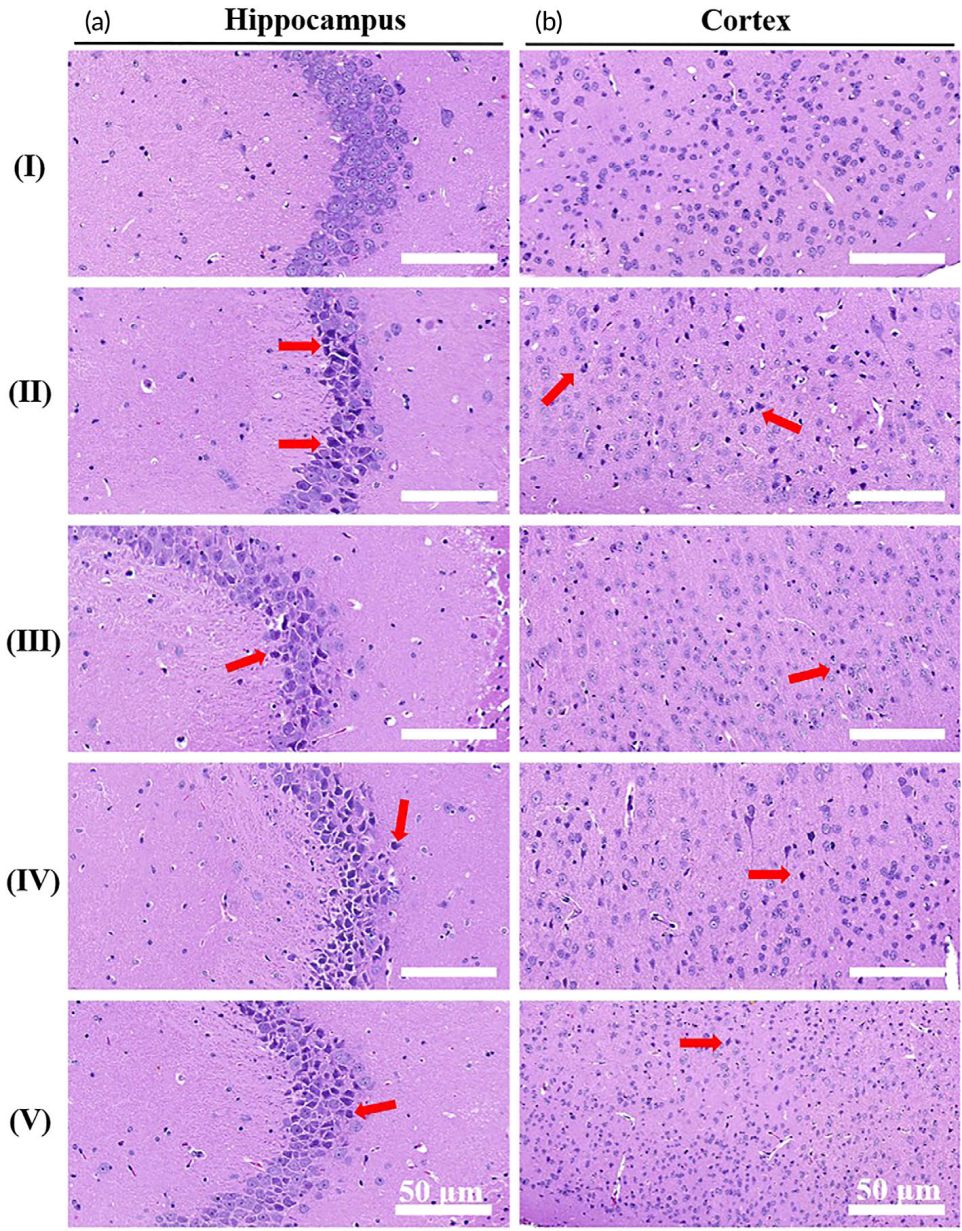
Morphological changes of (a) hippocampal and (b) cortex neurons were observed by using HE staining. (I) WT + PBS, (II) Tg + PBS, (III) Tg + RIF, (IV) Tg + RIF@PLA‐PEG‐Gd/Mal, (V) Tg + RIF@PLA‐PEG‐Gd/Mal‐RVG29. Scale bars: 50 μm (magnification: ×40)

Aβ has a direct cytotoxic effect on neurons, resulting in a large number of neuron loss and degeneration.^56^ Rifampicin was found to prevent Aβ(1–40)‐induced neurotoxicity on rat pheochromocytoma PC12 cells.^57^ Rifampicin pretreatment can protect PC12 cells from rotenone‐induced cell death. Rifampicin significantly inhibited rotenone‐induced apoptosis by reducing oxidative stress in mitochondria.^58^ An in vivo study showed that rifampicin attenuates MPTP‐induced neurodegeneration in nigrostriatal dopamine neurons of mouse brains.^59^ The above research results show that rifampicin has a neuroprotective effect in vitro and in vivo in mice. In this study, H&E staining was performed to evaluate the effect of RIF@PLA‐PEG‐Gd/Mal‐RVG29 on neurons in the brain of Tg mice (Figure 9). For WT + PBS group, the neuronal cells were neatly arranged, and the cell morphology was normal and uniformly distributed (Figure 9a(I),b(I)). However, Tg + PBS group revealed significant injuries including disordered and sparse cell arrangements, remarkable cell loss, karyopycnosis, and hyperchromatic nuclei in hippocampal and cortical neurons (Figure 9a(II),b(II)). Necrosis and loss of neurons were reduced in Tg mice treated with RIF, RIF@PLA‐PEG‐Gd/Mal, and RIF@PLA‐PEG‐Gd/Mal‐RVG29 (Figure 9a(III, IV, V),b(III, IV, V)).

### 3.9 In vivo toxicity studies of nanoparticles

Automatic biochemical analyzer was used to evaluate the toxicity of RIF@PLA‐PEG‐Gd/Mal and RIF@PLA‐PEG‐Gd/Mal‐RVG29 in each. We detected the creatinine value of mice in each group. There was no significant difference among groups in the creatinine value of mice (Figure S3). This result indicated that the biosafety of NPs is reliable in vivo.

## 4 CONCLUSION

In this work, we successfully designed and prepared rifampicin‐loaded brain‐targeted nanoparticles (RIF@PLA‐PEG‐Gd/Mal‐RVG29). The physical and chemical properties of these nanoparticles were revealed by investigating the drug loading, encapsulation efficiency, particle size, in vitro drug release, cytotoxicity, and distribution in the brain. Effects of RIF@PLA‐PEG‐Gd/Mal‐RVG29 on the improvement of cognitive function, Aβ deposition, synaptic ultrastructure, a synaptic protein, and changes in neuron morphology in APP/PS1 mice with AD were further explored. The prepared RIF@PLA‐PEG‐Gd/Mal‐RVG29 can improve the bioavailability of rifampicin due to its good biocompatibility, brain targeting, and controlled release drug. In vivo, our study showed that these nanoparticles can effectively improve cognitive impairment, reduced Aβ deposition and neuronal death, and promoted synaptic remodeling in AD mice. In addition, the prepared nanomedicine has the potential in diagnosis of AD and evaluation of drug efficacy through MRI. Thus, RIF@PLA‐PEG‐Gd/Mal‐RVG29 is a promising biodegradable material for AD treatment.

## AUTHOR CONTRIBUTIONS

Wei Bi, Rui Guo and Lihong Zhu designed the study. Lihong Zhu, Wei Bi and Rui Guo participated in the planning of the study and review of the data. Rui Guo was responsible for the design, performance testing and preparation of nanomaterials. Ruiyi Zhou performed the majority of experiments. Zhaohao Zeng, Rixin Luo and Jiawei Zhang assisted in carrying out Morris water maze experiment, In vivo real‐time imaging and In vivo MRI study. Lei Zhang provided technical support for the in vivo MRI study. Qunying Zhang coordinated the study. Ruiyi Zhou wrote the manuscript. Wei Bi, Rui Guo and Lihong Zhu revised the manuscript.

## CONFLICT OF INTEREST

There are no conflicts to declare.

## Supporting information


**Data S1** Supporting information

## Data Availability

Data available on request due to privacy/ethical restrictions.
