# Supplementary material for: Targeted brain delivery of RVG29‐modified rifampicin‐loaded nanoparticles for Alzheimer's disease treatment and diagnosis
Source: Bioeng Transl Med. 2022 Aug 26;7(3):e10395. doi: 10.1002/btm2.10395 (PMC9472014; doi:10.1002/btm2.10395)
Supplement: Supplementary file 1 — Data S1 Supporting information [file BTM2-7-e10395-s001.docx]

**Supporting Information**

**Targeted Brain Delivery of RVG29-Modified** **Rifampicin-Loaded Nanoparticles for Alzheimer** **Disease Treatment and** **Diagnosis**

Ruiyi Zhou^#,1^, Lihong Zhu^#,2^, Zhaohao Zeng^1^, Rixin Luo^1^, Jiawei Zhang^2^, Li Deng ^3^, Rui Guo ^3^, Lei Zhang^4^, Qunying Zhang^5^, Wei Bi ^*,1^

*^1^ Department of Neurology, The First Affiliated Hospital, Jinan University, Guangzhou, Guangdong 510632, China*

*^2^ Department of Pathophysiology, Key Laboratory of State Administration of Traditional Chinese Medicine of the People’s Republic of China, School of Medicine, Jinan University, Guangzhou, Guangdong 510632, China*

*^3^ Key Laboratory of Biomaterials of Guangdong Higher Education Institutes, Guangdong Provincial Engineering and Technological Research Center for Drug Carrier Development, Department of Biomedical Engineering, Jinan University, Guangzhou 510632, China*

*^4^ Department of Cerebrovascular Disease, The Fifth Affiliated Hospital of Sun Yat-sen University，Zhuhai , Guangdong 519000, China*

*^5^ Department of Cardilogy, The Fifth Affiliated Hospital of Sun Yat-sen University，Zhuhai , Guangdong 519000, China*

*****Correspondence to: *Wei Bi, Department of Neurology, The First Affiliated Hospital of Jinan University, Guangzhou 510630, PR China***,** *biwei4762@sina.com*

^＃^These authors contributed equally to this work.

**Supplementary methods.**

**1. Preparation of PLA-PEG-Gd**

100 mg of PLA-PEG-NH_2_ and 100 mg of DTPA were dissolved in 10 ml of DMSO. After adding a small amount of triethylamine, the reaction was carried out at 45 °C overnight. The reaction solution was dialyzed in a dialysis bag with a cut-off molecular weight of 3500 Da for 24 h to remove the impurities. The dialysate was freeze-dried to obtain PLA-PEG-DTPA. Then, 100 mg of PLA-PEG-DTPA and 200 mg of rhenium chloride hexahydrate were dissolved in 15 ml of water/dioxane (1:2v/v). The pH of the solution was adjusted to 6.0 ~ 6.5 by adding 0.1 M NaOH. The reaction mixture was stirred at 50 °C for 4 h. Finally, the reaction mixture was dialyzed in a dialysis bag (cut-off M.W. 1000 Da) for 24 h. Finally, PLA-PEG-Gd was prepared by freeze-drying the dialysate.

**2.** **The encapsulation efficiency and loading capacity**

The UV VIS spectrophotometer (UV, V-3100PC, Mapada, China) was used to evaluate the rifampicin of different concentration dissolved in DMSO. The absorbance was measured in the wavelength range of 350~600 nm, and the absorbance of the maximum absorption peak was taken as the standard curve. UV-vis spectra of RIF at various concentrations and the standard curve of RIF were showed in Fig. S1. After RIF@PLA-PEG-Gd/Mal and RIF@PLA-PEG-Gd/Mal-RVG29 were dissolved with DMSO and prepared into 1mg/ml solution. The absorbance of the two sample at 480.5 nm was measured by UV-Vis spectrophotometer, and calculated the concentration through standard curve. The encapsulation efficiency and loading capacity was determined by the following formula:

Encapsulation efficiency (%) = (Mass of rifampicin in NPs/Mass of NPs) × 100 Drug loading (%) = (practical load of rifampicin/theoretical load of rifampicin) ×100

**3. *In vitro* MRI study**

An aqueous solution of 3 g/L PLA-PEG-Gd/Mal (0.5400 mM Gd) was prepared and then gradually diluted into 8 samples of different concentrations, where the Gd concentration was 0.0042, 0.0084, 0.0169, 0.0338, 0.0675, 0.1350, 0.2700, 0.5400 mM, respectively. All the samples were scanned on a Gyroscan Intera 1.5 T magnetic resonance imaging (1.5T MRI, Philips Medical Systems, The Netherlands). T1-weighted spin-echo with the following parmeters: repetition time (TR) =1500 ms, echo time (TE) =96.191, 167.98, 293.32, 487.83, 752.57, 1032.6, 1259.3, 1430.2 ms, FS=1.5.

**4. Drug release assay**

The drug-release was study by a dialysis method. A dialysis bag containing 1 ml of 2 mg/ml Rifampicin @ PLA-PEG-Gd-RVG29 was immersed in a centrifuge tube containing 10 mL of PBS (0.01 M, pH = 7.4), and placed the centrifuge tube in incubator at 37 ℃and oscillate at 150 rpm. Rifampicin content of dialysate was detected by UV-Vis spectrophotometer at different time (0 h, 0.15 h, 0.5 h, 1 h, 1.5 h, 2.5 h, 3.5 h, 5 h, 7 h, 24 h). 1 mL of sample dialysate was taken at each time point, and then replaced with 1 ml PBS buffer into the dialysis bag. The cumulative release amount of Rifampicin from RIF@PLA-PEG-Gd-RVG29 was plotted against time.

**5. Toxicity studies**

Cell Counting Kit-8 (CCK8) assays was used to evaluate the toxicity of PLA-PEG-Gd/Mal, RIF@PLA-PEG-Gd/Mal and RIF@PLA-PEG-Gd/Mal-RVG29 in HT22 cells. HT22 cells were seeded in 96 well plates with a density of 5000 cells/well, and then cultured with growth medium (DMEM+10% FBS+100 IU/ml penicillin+10 IU/ml streptomycin) under 5% CO_2_ at 37 ℃. The plates were incubated to allow the cells to attach. The cells were added different concentrations of PLA-PEG-Gd/Mal, RIF@PLA-PE-Gd/Mal and RIF@PLA-PEG-Gd/Mal-RVG29, respectively. The plates were incubated for 24 h. 100 ml fresh medium (10% CCK8) were added into per well. The absorbance values were detected at 450 nm by Model Microplate Reader. After the morris water maze, the blood of mice was collected, and then the supernatant was collected by centrifugation. The sample concentration of each group was detected by Automatic Biochemical analysis (Chemray 800, Rayto Life and Analytical Sciences Co., Ltd.). The absorbance was measured by Mycotoxin Detector (Epoch, BioTeK), and then the creatinine concentration was converted.

**6. Cellular uptake studies**

1 × 10^5^ bEnd.3 cells were plated in a six-well plate and then adhered overnight. Both of targeted and non-targeted rifampicin-loaded nanoparticles were diluted with DMEM to 30 μg/ml, and then incubated with the cells for 4 h at 37 °C. The cells were trypsinized, washed thoroughly with PBS, and resuspended in staining buffer. Confocal laser scanning microscope (CLSM, LEICA, SP8, Germany) was used to observe the fluorescence state of cells and evaluate the *in vitro* targeting effect of RVG29-targeted drug delivery system.

**7. Morris water maze (MWM)**

Behavioral tests were performed on the second day after the last dose. Morris water maze experiment aims to assess the spatial learning and memory abilities of mice. Morris water maze consists of a basin, underwater platforms, an automatic image acquisition and processing system composed of cameras, video recorders, monitors and analysis software. The basin is a circular drum with a diameter of 120 cm and a height of 50 cm. The water in the basin was opaque by adding white powder with the temperature of 23 ± 1 ℃. The MWM experiment includes a 7-day directional navigation experiment and a 1-day space exploration experiment. Before the test, these mice were allowed to swim freely in the water for 1 min, and then left on the platform for 10 s to adapt to the environment.

Directional navigation experiment: The mice entered the water from 4 different quadrants each day, and the order of entering the initial quadrant was different each time. If the mice climbed onto the platform and stayed for more than 3 s within 60 s after entering the water, the recording time was the latency time. If the mice did not find the platform within 60 s, the latency time was recorded as 60s. Then the mice were guided to rest on the platform for 10 s before next experiments. The experiment lasted for 7 days.

Space exploration experiment: The next day after the completion of the directional navigation experiment, the distant quadrant from the platform quadrant was selected as the entry point for the mice. Superfluous platforms were removed. The mice were put into the water for 60s, and the platform number they crossed and the time they stayed on the platform quadrant were recorded.

**8. Transmission electron microscopy**

Hippocampal CA1 tissue of mouse was fixed with pre-cooled 2.5% glutaraldehyde, followed by dehydration, embedding, and solidification. This tissue was sectioned and then stained with uranyl acetate and lead citrate. Synapse structures in hippocampal CA1 area were observed under transmission electron microscope.

**9 ^1^H NMR characteristic of PLA-PEG-DTPA**

The ^1^H NMR results of PLA-PEG-DTPA are shown in Fig. S2. The chemical shift δ5.2 is attributed to the parahydrogen on the lactic acid skeleton. δ3.6 is attributed to the proton of -CH_2_CH_2_- on the ethylene glycol skeleton. δ3.1~2.7 is attributed to the proton of -CH_2_-, which is the characteristic peak of the DPTA structure. δ1.9 is attributed to the proton of -CH_2_CH_2_- on the DTPA structure. δ1.2~1.4 is attributed to the proton of -CH_3_ on the lactic acid skeleton. ^1^H NMR result further confirms that PLA-PEG-DTPA was successfully prepared.

**Supporting Figures**

**Fig. S1** ^1^H NMR result of PLA-PEG-DTPA.

**Fig. S2** UV-vis spectra of RIF at various concentrations and the standard curve of RIF.





**Fig. S3** **The effect of RIF@PLA-PEG-Gd/Mal-RVG29 on renal function of APP/PS1 mice. n = 3 per group.**
